# Cell‐type proteomic and metabolomic resolution of early and late grain filling stages of wheat endosperm

**DOI:** 10.1111/pbi.14203

**Published:** 2023-12-04

**Authors:** Shuang Zhang, Arindam Ghatak, Mitra Mohammadi Bazargani, Hannes Kramml, Fujuan Zang, Shuang Gao, Živa Ramšak, Kristina Gruden, Rajeev K. Varshney, Dong Jiang, Palak Chaturvedi, Wolfram Weckwerth

**Affiliations:** ^1^ Molecular Systems Biology Lab (MOSYS), Department of Functional and Evolutionary Ecology University of Vienna Vienna Austria; ^2^ National Technique Innovation Center for Regional Wheat Production/Key Laboratory of Crop Ecophysiology Ministry of Agriculture/Nanjing Agricultural University Nanjing China; ^3^ Vienna Metabolomics Center (VIME) University of Vienna Vienna Austria; ^4^ Agriculture Institute Iranian Research Organization for Science and Technology Tehran Iran; ^5^ Department of Systems Biology and Biotechnology, National Institute of Biology Ljubljana Slovenia; ^6^ State Agricultural Biotechnology Centre, Centre for Crop and Food Innovation, Food Futures Institute Murdoch University Murdoch WA Australia

**Keywords:** wheat, proteomics and metabolomics, aleurone, sub‐aleurone, starchy endosperm, endosperm transfer cells

## Abstract

The nutritional value of wheat grains, particularly their protein and metabolite composition, is a result of the grain‐filling process, especially in the endosperm. Here, we employ laser microdissection (LMD) combined with shotgun proteomics and metabolomics to generate a cell type‐specific proteome and metabolome inventory of developing wheat endosperm at the early (15 DAA) and late (26 DAA) grain‐filling stages. We identified 1803 proteins and 41 metabolites from four different cell types (aleurone (AL), sub‐aleurone (SA), starchy endosperm (SE) and endosperm transfer cells (ETCs). Differentially expressed proteins were detected, 67 in the AL, 31 in the SA, 27 in the SE and 50 in the ETCs between these two‐time points. Cell‐type accumulation of specific SUT and GLUT transporters, sucrose converting and starch biosynthesis enzymes correlate well with the respective sugar metabolites, suggesting sugar upload and starch accumulation via nucellar projection and ETC at 15 DAA in contrast to the later stage at 26 DAA. Changes in various protein levels between AL, SA and ETC support this metabolic switch from 15 to 26 DAA. The distinct spatial and temporal abundances of proteins and metabolites revealed a contrasting activity of nitrogen assimilation pathways, e.g. for GOGAT, GDH and glutamic acid, in the different cell types from 15 to 26 DAA, which can be correlated with specific protein accumulation in the endosperm. The integration of cell‐type specific proteome and metabolome data revealed a complex metabolic interplay of the different cell types and a functional switch during grain development and grain‐filling processes.

## Introduction

Wheat (*Triticum aestivum* L.) is one of the major sources of the human diet globally. Given the rapid growth of the world's population, there is an urgent need to increase wheat yield. In cereals, energy is mainly stored in the endosperm of the grain as starch, protein and lipid, accounting for an estimated 42.5% of the global calories of the human diet. Based on the hardness of the grain, wheat can be divided into soft, hard and durum wheat varieties (Medina‐Rodríguez *et al*., 2009). Desirable grain‐filling traits for bread wheat are higher rates of starch production for yield and high protein content for bread‐making (Dupont, 2008).

Grains primarily comprise embryo and endosperm, which play important roles in seed germination and subsequent plant growth and development. The endosperm contains reserve substances to supply nutrients for subsequent plant growth based on wheat yield and quality. The endosperm comprises aleurone (AL), sub‐aleurone (SA), starchy endosperm (SE) and endosperm transfer cells (ETCs), each having different spatial–temporal physiological and molecular mechanisms (Olsen, 2001; Zheng and Wang, 2014). During wheat grain endosperm development, the outermost layer of endosperm cells differentiates into highly specialized tissue—aleurone (AL; Wang *et al*., 1995). AL contain various hydrolases, which hydrolyse the starch and seed storage protein (SSP) stored in endosperm during germination. Sub‐aleurone (SA) is several layers between AL and SE, primarily acting as a transition stage. SSPs are highly accumulated in SA, characterized by secondary wall ingrowth (Hermans *et al*., 2021; Jacobs *et al*., 2018). SE is the main cell type of the endosperm, accumulating starch and seed storage protein. ETCs are surrounding the cavity fluid, forming the endosperm cavity, also named ‘modified aleurone’. It is a highly specialized cell with secondary wall ingrowth, which transports the substrates and signal molecules from cavity fluid to the endosperm. ETCs are upstream of grain‐filling substrate transportation, potentially controlling regulatory processes of the grain‐filling (Olsen, 2001, 2004; Shabrangy *et al*., 2018).

Structurally it has been observed that in the developing endosperm of wheat grain photosynthetic assimilates are transferred from the SE‐CC complex (sieve elements and companion cells) to the nucellar projection transfer cells through the crease phloem into the endosperm cavity (Wang and Fisher, 1995). After being unloaded into the endosperm cavity, assimilates enter the wheat endosperm via two pathways: one is transported to the endosperm through the AL; the other is through the transfer cells around the endosperm cavity radially to the endosperm. As the modified aleurone, ETCs exhibited an irregular shape, and notable intercellular gaps were observed. These cells are developed approximately 2–3 days before the advancement of AL, ultimately succumbing to degeneration after the filling process (Xiong *et al*., 2013). Due to the same structural characteristics, substrates are more easily transported to the AL by endosperm transfer cells than other cell types. For example, it was found that the level of ^15^N‐labelled glutamine during 17 days after anthesis (DAA) in the inner endosperm was higher than that in the outer endosperm, indicating that amino acids can be transported radially from the endosperm cavity to the endosperm tissue through transport cells (Moore *et al*., 2016). However, it is unclear how these two pathways regulate the grain‐filling process and which pathway functions as the main pathway in different grain‐filling stages. Furthermore, photosynthetic assimilates from the cavity via ETCs are transferred to the inner endosperm through the symplast (Wang and Fisher, 1995). Therefore, it is important to investigate the physiological and molecular mechanisms of the grain‐filling process. Although different proteomic studies on wheat grain development have been conducted, these investigations primarily focused on a single organ or tissue such as whole seeds, endosperm and embryo (He *et al*., 2015; Yu *et al*., 2016; Zhang *et al*., 2021). This approach does not provide an overall understanding of the metabolic and regulatory networks occurring in the different cellular types of the grain.

In the present study, we have undertaken laser microdissection sampling combined with multiomics approaches to identify the abundant proteins and metabolites in the different cell types of wheat endosperm (AL, SA, SE, ETCs). Based on our previous study on grain development (Zhang *et al*., 2021) and the accumulation pattern of the assimilates, we selected two decisive time points which are early/mid (15 DAA) and late stage (26 DAA) of the grain filling. We established a dynamic landscape of the biochemical processes occurring during development in different cell types of the endosperm. Possible key metabolic steps relevant to starch biosynthesis and the regulation of amino acid metabolism are discussed. Furthermore, we also generated a reference map of the endosperm proteins and metabolites, which will facilitate future breeding studies, including the effect of environmental conditions on the grain development and composition/quality of the mature grain.

## Materials and Methods

### Plant material and growth conditions

The elite Chinese bread wheat (*Triticum aestivum* L.) cultivar Yangmai 16, a medium gluten wheat variety with a large spike and high yield, was selected for this study (Zhang *et al*., 2021). The plants were grown in a controlled condition (12 h of light, 120 μmol/m^2^/s, 23°C during daytime, 20°C at night, 60% humidity). The soil mixture consisted of three parts of potting ground (peat, humus), 2 parts of sand, 1 part of styromull and 0.1% NPK was added as initial fertilizer and no pesticides were used. Plants were watered periodically. The main culm spikes were tagged upon anthesis, and the grains from the labelled spikes were harvested during 15 and 26 days after anthesis (DAA). Grains were sampled from the four spikelets at the centre of each spike. Further harvested grains were dissected, and the sections from the central region were excised using a razor blade (Shabrangy *et al*., 2018). The samples were then transferred to a silicon mould (Plano, Wetzlar, Germany), and cryogel (Plano) was added and immediately frozen in liquid nitrogen to stop all enzymatic activity stored at −80°C until further microdissection. Before laser microdissection (LMD), a series of 20 μm sections were cut and immediately mounted on SuperFrost® Plus sides (Thermo Scientific, Karlsruhe, Germany), which were stored in a Falcon tube containing silica gel to absorb moisture according to Shabrangy *et al*. (2018). After 30 min, these dry cryosections were used for LMD cuts.

### Measurement of physiological parameters

**Figure 1:**
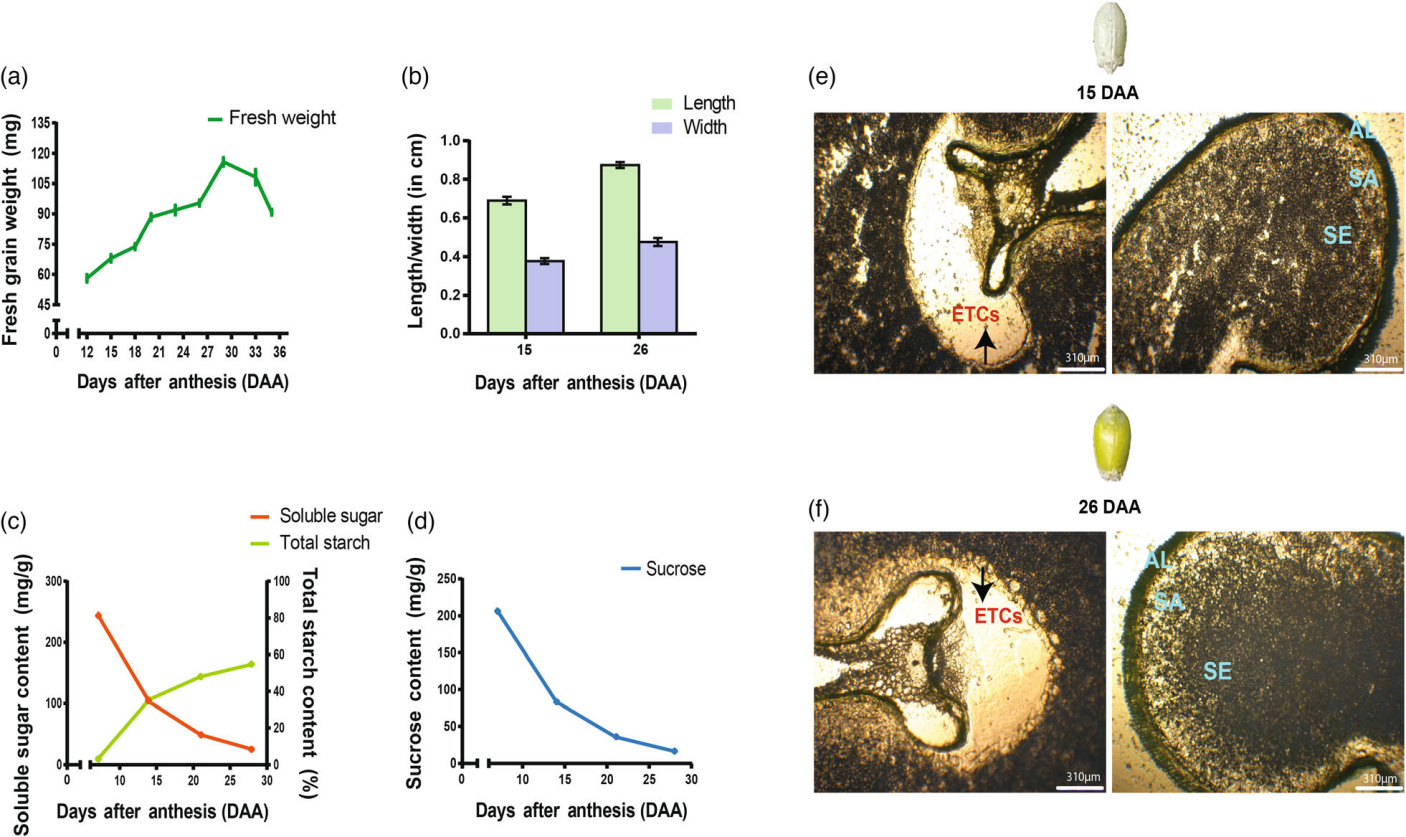
Dynamics of grain development and sucrose, starch and soluble sugar contents. (a) Changes in fresh weight of the developing grains. At least 70 grains were analysed at each stage. (b) Changes in grain length and width between 15 and 26 DAA. Error bars represent the SD of three replicates. (c) Soluble sugar and total starch content of the developing grains. (d) Sucrose content of the developing grains. The error bars indicate the standard error from four biological replicates. (e) Endosperm slice observed using Leica® laser micro dissecting microscope (LMD6500), magnification 310 μm from 15 DAA. (f) Endosperm slice observed using Leica® laser micro dissecting microscope (LMD6500), magnification 310 μm from 26 DAA. AL, aleurone; DAA, days after anthesis; ETCs, endosperm transfer cells; SA, sub‐aleurone; SE, starchy endosperm.

Yangmai 16 grains were collected at different development stages (12–35 DAA) to determine the fresh weight (Figure 1a; Table S1). Additionally, the length and width of four grains in three replicates were recorded at 15 and 26 DAA (Figure 1b; Table S1).

### Sugars, soluble sugars and total starch content in developing whole grains

The content of soluble sugars and sucrose content were determined in developing whole grains according to the method established by Dubois *et al*. (1956) and Khanzada *et al*. (2022) (Figure 1c,d; Table S1). In brief, developing grains were soaked in 5 mL 80% ethanol, followed by a water‐bath incubation at 80 °C for 10 min and then centrifugation for 10 min at 3000 ***g***. This extraction step was repeated twice, and the final volume was adjusted to 5 mL with 80% ethanol. The total soluble sugars were determined by colorimetric quantification at 620 nm using the anthrone reagent method. Sucrose content was estimated by colorimetric quantification at 480 nm with resorcinol assay.

The total starch content was measured using Ewers polarimetric method (STN EN ISO 10520; 1997), which is based on the partial acid hydrolysis of starch followed by measurement of the optical rotation of the resulting solution (Šimora *et al*., 2019; Figure 1c; Table S1). According to this method, 1 g of homogenized sample was dissolved in 10 mL 0.33 m HCl solution and incubated for 10 min in boiling water. After cooling, 0.5 mL 30% (m/v) zinc sulfate and 0.5 mL 15% (w/v) potassium ferrocyanide were added to the sample and mixed well. Finally, the sample was diluted with distilled water to the final volume of 25 mL and measured using polarimeter. The optical rotations of all samples were measured at 20 °C using a sample cell of 200 mm optical path length.

### Laser micro dissection (LMD)

The Leica laser® micro dissecting microscope (LMD6500) coupled with LMD Software was used for microdissection and capturing images (Figure 1e,f). The aleurone (AL), sub‐aleurone (SA), starchy endosperm (SE) and endosperm transfer cells (ETCs) were separated by a 355 nm laser (Figure S1). The regions dissected by laser were removed by gravity into the corresponding lids of precooled PCR tubes placed under the slide. In total, 60 sections were dissected to collect the cell types, i.e. aleurone (AL; 0.085 g), sub‐aleurone (SA; 0.091 g) and starchy endosperm (SE) (0.092 g), while 100 sections were collected for endosperm transfer cells (ETCs; 0.073 g). The dissected samples were stored at −80°C before the protein extraction. Three biological replicates for each cell types were analysed at each developmental time point to minimize experimental errors.

### Proteomics analysis

For proteomics analysis, different cell types were freeze‐dried in liquid N_2_ and ground for 2 min in a shaking mill using steel balls (2 mm diameter). The proteins were extracted, pre‐fractionated (40 μg of total protein were loaded onto the gel (1D SDS‐PAGE)), trypsin digested and desalted (using a C18 spec plate) according to a previously described method (Chaturvedi *et al*., 2013). Prior to mass spectrometric measurement, the tryptic peptide pellets were dissolved in 4% (v/v) acetonitrile, 0.1% (v/v) formic acid. One μg of each sample (three replicates for each cell types) was loaded on a C18 reverse‐phase column (Thermo Scientific, EASY‐Spray 500 mm, 2 μm particle size). Separation was achieved with a 90 min gradient from 98% solution A (0.1% formic acid in high purity water (MilliQ)) and 2% solution B (90% ACN and 0.1% formic acid) at 0 min to 40% solution B (90% ACN and 0.1% formic acid) at 90 min with a flow rate of 300 nL/min. nESI‐MS/MS measurements were performed on Orbitrap QExactive (Thermo Fisher Scientific, Bremen, Germany) with the following settings: Full scan range 350–1800 *m*/*z* resolution 120 000 max. 20 MS2 scans (activation type CID), repeat count 1, repeat duration 30 s, exclusion list size 500, exclusion duration 30 s, charge state screening enabled with the rejection of unassigned and +1 charge states, minimum signal threshold 500.

### Peptide and protein identification

Raw data were searched with the SEQUEST algorithm present in Proteome Discoverer version 1.3 (Thermo, Germany) described previously (Ghatak *et al*., 2020). The UniProt database for wheat containing the annotation of 136 866 genes was used for identification. In parallel, the raw data were also searched against the IWGSC (International Wheat Genome Consortium Sequences) database containing annotation of 106 914 genes (Table S2). Peptides were matched against these databases plus decoys, considering a significant hit when the peptide confidence was high, which is equivalent to a false discovery rate (FDR) of 1%, and the Xcorr threshold was established at 1 per charge (2 for +2 ions 3 for +3 ions, etc.). The variable modifications were set to acetylation of the N‐terminus and methionine oxidation, with a mass tolerance of 10 ppm for the parent ion and 0.8 Da for the fragment ion. The number of missed and nonspecific cleavages permitted was two. There were no fixed modifications, as dynamic modifications were used. All the MS/MS spectra of the identified proteins and their meta‐information from both databases were further uploaded to the PRIDE repository. Project accession: PXD035848 (see Data availability Statement).

The identified proteins were quantitated based on total ion count and normalized using the normalized spectral abundance factor (NSAF) strategy (Paoletti *et al*., 2006).

### Metabolite extraction, profiling and peak annotation

Metabolite extraction, sample derivatization and GC‐TOF‐MS (gas chromatography coupled with time‐of‐flight mass spectrometry) were performed as described previously (Ghatak *et al*., 2022; Zhang *et al*., 2021). Data analysis was performed using ChromaTof (Leco) software. Briefly, endosperm cell types (aleurone (AL), sub‐aleurone (SA), starchy endosperm (SE) and endosperm transfer cells (ETCs)) were freeze‐dried and grounded to a fine powder using mortar pestle for metabolite extraction. The representative chromatograms of each cell type of different samples were used to generate a reference peak list, and all other data files were processed against this reference list. Deconvoluted mass spectra were matched against the in‐house mass spectral library. Peak annotations and peak integrations were checked manually before exporting peak areas for relative quantification (De La Harpe *et al*., 2020). Metabolite amounts are given in arbitrary units corresponding to the peak areas of the chromatograms. The heatmap was generated using the R program. Abundance levels for selected metabolites are the mean and standard deviation from three biological replicates for each cell type (AL, SA, SE, ETCs) and developmental stage (15 and 26 DAA).

### Bioinformatics, statistics and functional annotation

The proteome and metabolome data were subjected to multivariate (principal component analysis (PCA)) analysis, which was performed using the statistical toolbox COVAIN in Matlab (Sun and Weckwerth, 2012) and R program (package ggplot2). For K‐means clustering analysis, proteins were chosen only if present in all three biological replicates of at least one cell type and developmental stage. The Venn diagrams were produced using a Web tool (http://bioinformatics.psb.ugent.be/webtools/Venn/). Functional descriptions of wheat sequences were assigned according to a study performed by Ghatak *et al*. (2020) and Ramsak *et al*. (2014).

To indicate the protein accumulation pattern at different cell types, the relative protein ratios of samples of each cell type against 15DAA were calculated (Yu *et al*., 2016). Differentially expressed proteins (DEPs) between developing stages (15 and 26 DAA) for each cell type (aleurone, sub‐aleurone, starchy endosperm and endosperm transfer cells) were identified considering two conditions: |log_2_FC| ≥ 1.5 and *P*‐value < 0.05. Log transformed values for DEPs are represented by heat maps generated using ClustVis (Metsalu and Vilo, 2015) and COVAIN in Matlab. All the identified proteins were categorized into functional groups to allow a functional view of the proteome in each cell type of the developing endosperm. Metabolic pathways were generated in‐house using MapMan classifications (Thimm *et al*., 2004).

## Results

### Morphology, soluble sugars, sucrose, total starch content of developing wheat grain

Fresh weight (mg per grain) of developing grain increased with maturity until 30 DAA and then declined (Figure 1a). An increase in the fresh weight could be explained on the basis of dry matter accumulation and the increase in the water content of the grains. Since cavity fluid disappears after 28 DAA (Zhang *et al*., 2021), indicating the start of the maturity stage, fresh weight was bound to decrease after attaining a peak value at 30 DAA. To understand the regulation of assimilate import within the wheat grain, we measured the sucrose, soluble sugars and total starch content levels of the developing grains (7–28 DAA) (Figure 1 c, d). The soluble sugars and sucrose content increased immediately after the onset of anthesis, reaching a maximum concentration at 7 DAA and decreasing until 28 DAA. Hereafter, the level tended to remain almost constant. The grains exhibit a small quantity of starch at the 7 DAA. Subsequently, it increased rapidly until maturity (30 DAA). Active starch synthesis commenced from 14 DAA and remained at maximum until 28 DAA.

In the study performed by Zhang *et al*. (2021), 15 and 26 DAA have been identified as two important transition time points during the grain‐filling process, in which 15 DAA is the medium milk stage and 26 DAA is the hard dough stage. Additionally, the spatial distribution of seed storage proteins (SSPs) first appeared at 14 DAA, and protein accumulation started rapidly (Tosi *et al*., 2009). Structurally, from 24 days post‐anthesis (DPA), the protein matrix surrounding the starch granules is visible instead of individual protein bodies (PBs; Yang *et al*., 2019). Considering the regulation of the assimilates during the grain‐filling process, in the present study, 15 DAA was selected as the early/mid‐grain filling time point, and 26 DAA was chosen as the late grain filling time point. All the recorded observations are summarized in Table S1.

### Cell‐specific proteome characterization of aleurone, sub‐aleurone, starchy endosperm and endosperm transfer cells using laser microdissection (LMD) and shotgun proteomics

**Figure 2:**
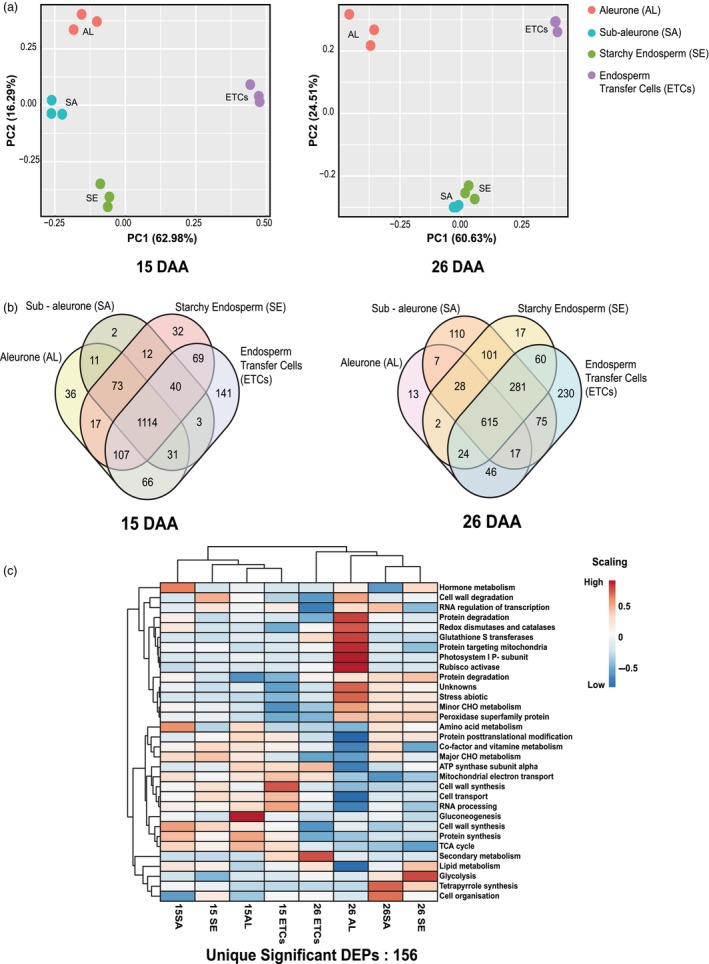
Regulation of the proteome in different cell types of the wheat endosperm. (a) Principal component analysis (PCA) of the protein profiles of different cell types during 15 and 26 DAA. Three independent biological replicates were used for proteomic analysis. (b) Venn diagrams represent complete proteome identification from different cell types of developing grain (15 and 26 DAA). (c) Heat map representing differentially expressed proteins (DEPs) in different cell types during 15 and 26 DAA of wheat grain filling. AL, aleurone; DAA, days after anthesis; ETCs, endosperm transfer cells; SA, sub‐aleurone; SE, starchy endosperm.

For protein identification, the raw data were searched against wheat UniProt and IWGSC (International Wheat Genome Sequencing Consortium) databases (Table S2). In the present study, the UniProt database was considered for the functional interpretation of the identified proteins because of its accurate, consistent and rich annotations of protein names, descriptions and biological functions (UniProt, 2021). Wheat genome sequencing and functional annotation were recently completed (International Wheat Genome Sequencing, 2018; Shewry, 2009); therefore, not many online freeware is available for the proteomics community that can be applied to wheat datasets (Vincent *et al*., 2022). However, we detected 2686 *Traes* identifiers mapped for *T. aestivum* functional overview (see Materials and methods; Table S2). At this stage, we decided to proceed with the UniProt database because of a consistent functional description of the peptide and respective protein sequences in contrast to the IWGSC database. From all the detected peptides from the UniProt database, 1803 proteins were further selected considering proteins present in all three biological replicates at least in one cell type (Table S3). Principal component analysis (PCA) was performed using the protein NSAF scores. The first principal component separated the endosperm cell types, PC1, which accounted for 62.98% and 60.63% in the 15 and 26 DAA, respectively (Figure 2a; Table S4). Positive loadings of PC1 represented proteins with abundance in the ETCs, while negative loadings represented higher abundances in the AL (Table S4). In 15 DAA, the highest negative loadings included proteins involved in amino acid metabolism, CHO metabolism, protein synthesis and degradation and serpin (serine protease inhibitor). The proteome regulation in the negative loadings of 26 DAA includes proteins involved in RNA processing and regulation of transcription, showing higher abundances. The highest positive loadings in 15 DAA include proteins involved in cell wall degradation, auxin regulation, lipid metabolism, protein degradation and RNA regulation of transcription. Similarly, in 26 DAA, the highest positive loadings revealed proteins involved in cell wall precursor synthesis (late embryogenesis abundant protein D‐34), protein synthesis and targeting. In 26 DAA, less variance was observed between SA and SE, indicating that in the course of development, starchy endosperm becomes the central endosperm cell in the wheat grain (Figure 2a).

The pronounced changes in the proteome of different cell types (AL, SA, SE, ETCs) during the developmental stages (15 and 26 DAA) is illustrated by a Venn diagram (Figure 2b; Table S5). In total, 1114 and 615 proteins were commonly identified in all cell types at 15 and 26 DAA, respectively. Notably, two unique proteins (elongation factor EF‐2 and asparaginase) were only identified in SA at 15 DAA (Figure 2b). Elongation factor EF‐2 is crucial for protein synthesis, and asparaginase is an enzyme that catalyses the hydrolysis of asparagine to aspartic acid (Table S5). The carbamoyl‐phosphate synthase large chain was identified in AL and SA at 15 and 26 DAA, which is involved in amino acid degradation. At 26 DAA, two isoforms of cystathionine gamma‐lyase were identified, especially in AL, related to cysteine release during the grain‐filling process.

### Differentially expressed proteins (DEPs) in different cell types of endosperm during grain filling stages.

A cutoff on log_2_ (fold change) of 1.5 and *P*‐value < 0.05 was used to identify significant changes in the protein abundance and classify it as a differentially expressed protein (DEP). These DEPs were identified by comparing each cell type in developing stages (15 DAA and 26 DAA), that is, AL 15 DAA vs AL 26 DAA, SA 15 DAA vs SA 26 DAA, SE 15 DAA vs SE 26 DAA, and ETCs 15 DAA and ETCs 26 DAA (Table S6). In AL 15 DAA vs AL 26 DAA, a total of 67 DEPs were identified in the AL, 31 in SA, 27 in the SE and 50 in ETCs. This analysis aimed to determine the overall enrichment trend of the specific functional categories during the grain‐filling process, as shown in Figure 2c. DEPs were categorized according to the MapMan plant functional ontology (Ghatak *et al*., 2020; Ramsak *et al*., 2014). In total, 156 unique DEPs were identified across the comparison between the cell types during 15 and 26 DAA (Figure 2c, Table S6). Interestingly, DEPs involved in glycolysis were highly abundant in SE at 26 DAA compared to 15 DAA, indicating that carbohydrate metabolism was relatively active in SE at the late grain‐filling stage (Figure 2c). Glycolysis is also involved in amino acid biosynthesis because it converts glucose‐1‐phosphate (Glc‐1‐P) to a series of metabolites, which can efficiently be used for starch and protein storage in the cereal endosperm (Yu and Wang, 2016).

### Functional annotation of the proteome in different cell types of endosperm during early and late grain‐filling stages

Functional categorization of the identified proteins was performed according to Ghatak *et al*. (2020) and Ramsak *et al*. (2014). Functional distribution of the total proteome in different endosperm cell types (AL, SA, SE and ETCs) in early/mid (15 DAA) and late (26 DAA) grain filling stages are depicted via heat map biclustering by summing up the total NSAF score of the functional categories (Chaturvedi *et al*., 2013; Ghatak *et al*., 2020; Figure S1; Table S7). Comparing all the endosperm cell types at different time points, proteins involved in amino acid metabolism showed increased levels in the SE at 26 DAA.

Interestingly, proteins involved in nitrate metabolism (such as glutamate synthase; GOGAT) were first highly accumulated in AL during the early/mid‐grain filling stage (15 DAA) but then turned to ETCs in 26 DAA, indicating an active cell type switch during the grain‐filling process (Figure S1; Table S7). In developing wheat grains, NH^+^_4_ is mainly assimilated through the combined action of glutamine synthetase (GS), glutamate synthase (GOGAT) and glutamate dehydrogenase (GDH; Wei *et al*., 2021). In the present study, GDH activity was high in 15 DAA and decreased rapidly in 26 DAA, that is, during the intensive filling of grains with storage compounds. These observations may indicate that GDH participates in anabolic processes in which the final product is a protein (Kwinta *et al*., 1999).

The sub‐categories of the protein function related to protein synthesis showed enhanced regulation in the outer endosperm (AL and SA) in both early/mid (15 DAA) and late (26 DAA) grain‐filling stages. In contrast, the proteins involved in post‐translational modification were highly regulated in the inner endosperm (SE and ETCs; Figure S1; Table S7). Furthermore, the proteins involved in the transport of sugar, metal and amino acid showed increased levels in ETCs at the early/mid‐grain filling stage (15 DAA), then turned to AL and SA during the late grain filling stage (26 DAA; Figure S1; Table S7).

For the four dissected cell types of the endosperm, K means cluster analysis of proteins in different grain‐filling stages (15 DAA and 26 DAA) was performed by the COVAIN toolbox, revealing spatially coordinated proteome changes in different cell types of endosperm during the grain development. K numbers were different according to the grain‐filling stages. The k was 35 clusters for 15 DAA and 15 for 26 DAA (Table S8).

In 15 DAA, clusters 12 and 25 showed proteins with more abundance in AL and a gradual decrease in the inner endosperm cell types. Two isoforms of pyruvate phosphate dikinase (PPDK) were found, playing an important role in starch synthesis and energy supply. In SE, clusters 5, 16 and 22 demonstrated proteins with high levels in 15 DAA (Table S8). Serine/threonine kinase and vacuolar protein sorting 35 showed significantly increased levels in SE. Similarly, in 26 DAA (clusters 4 and 7), vacuolar protein sorting 35 and 27 demonstrated increased levels in SE and ETCs (Table S8). Vacuolar protein sorting‐associated (Vps) is part of the Endosomal Sorting Complex Required for Transport (ESCRT) that performs the topologically unique membrane bending and scission reaction away from the cytoplasm. They are primarily required to transport membrane‐associated enzymes (Cai *et al*., 2014). In SA proteins binned in the cluster, 13 demonstrated increased levels in 26 DAA compared with 15 DAA (Table S8). Protein transport SEC13‐like protein was highly abundant in SA, which is involved in protein trafficking from the endoplasmic reticulum to the Golgi apparatus and nucleo‐cytoplasmic traffic (Niu *et al*., 2014). The increased protein level in 26 DAA indicates that the protein synthesis activity was high in SA. This explains why SA has higher protein concentrations‐ and less variance is observed between SA and SE during the late grain‐filling stage. In ETCs, clusters 8 and 26 demonstrated the enhanced regulation of the protein, such as late embryogenesis abundant protein D‐34 in 15 DAA. Three isoforms of vesicle‐associated membrane protein‐associated were identified with a similar pattern. Similarly, clusters 17, 33 and 35 also showed proteins with increased levels in ETCs (Table S8). These results indicated that the amino acid biosynthesis and protein modification were highly active in ETCs at 15 DAA, consistent with the hypothesis that ETCs are upstream of the assimilates transportation in the endosperm.

To indicate the protein accumulation pattern during the grain‐filling process among different cell types of the endosperm (AL, SA, SE and ETCs), we consider the protein expression of the samples obtained at 15 DAA as a reference. The relative protein expression ratios at 26 DAA against those at 15 DAA (26 DAA/15 DAA) were used to determine the abundance of a protein in each cell type (Yu *et al*., 2016; Figure S2; Table S9).

### Dynamic metabolome changes in the developing wheat endosperm

**Figure 3:**
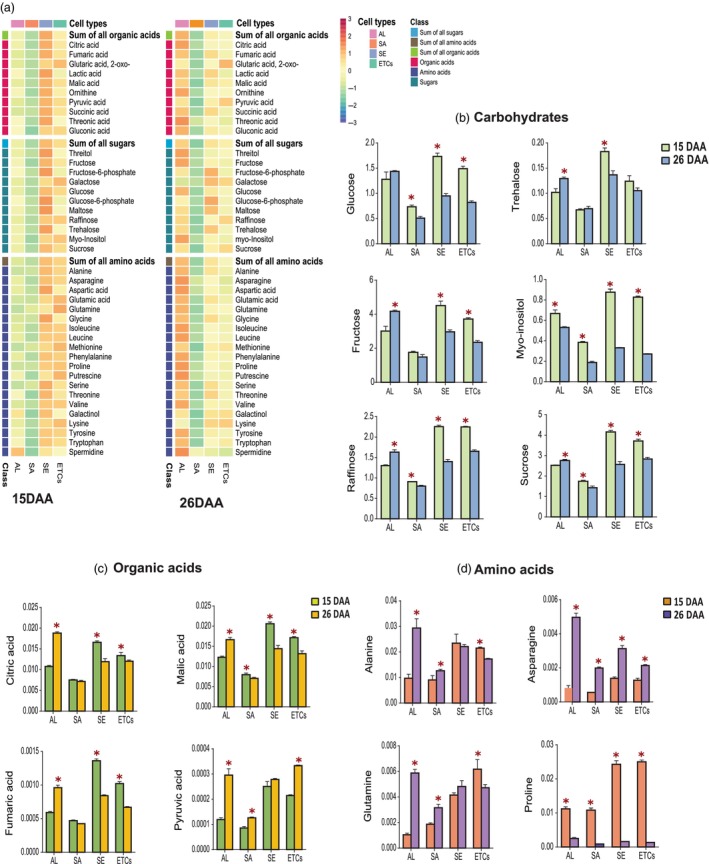
Regulation of the metabolome in different endosperm cell types during 15 and 26 DAA. (a) Classification of metabolites identified in different cell types during 15 and 26 DAA. (b) The bar graph represents the accumulation pattern of the selected carbohydrates. (c) The bar graph represents the accumulation pattern of the selected organic acids. (d) The bar graph represents the accumulation pattern of the selected amino acids. The error bars indicate the standard error from three biological replicates (*P‐value < 0.05). AL, aleurone; DAA, days after anthesis; ETCs, endosperm transfer cells; SA, sub‐aleurone; SE, starchy endosperm.

Metabolome profiling of four different cell types (AL, SA, SE, ETCs) of the developing endosperm (15 and 26 DAA) was conducted using GC‐time‐of‐flight (TOF)‐MS. In total, we identified 41 metabolites normalized by internal standard and fresh weight (Ghatak *et al*., 2022; Zhang *et al*., 2021). The identified metabolome was classified into carbohydrates, amino acids and organic acids (Figure 3; Table S10). Endosperm development is tightly linked to carbohydrate metabolism. In the present study, most of the identified carbohydrates (such as sucrose, glucose, fructose and myo‐inositol) were highly accumulated in SE and ETCs during 15 DAA compared with 26 DAA (Figure 3). Sucrose plays an important role in the regulation of the C/N balance during grain development and can also be used to synthesize starch (Weschke *et al*., 2000). Glucose and fructose are both products of photosynthesis, which demonstrated higher accumulation during 15 DAA. Myo‐inositol is responsible for oligosaccharides synthesis, phosphate storage and hormone transportation (Karner *et al*., 2004; Sharma *et al*., 2020). Similarly, enzymes involved in carbohydrate interconversion (like GLA, BF, BFF and SUSy) showed higher accumulation in the inner endosperm (SE and ETCs) in 15 DAA (Figure 3), which agrees with our proteomics analysis. The accumulation of carbohydrates in the inner endosperm at 15 DAA indicated energy and carbon storage as the active process during the early/mid endosperm development.

Glycolysis, the TCA cycle and the mitochondrial electron transport chain are essential to provide energy to many cellular functions. We identified several organic acids, including fumaric acid, citric acid and malic acid, all of which play important roles in the TCA cycle. These organic acids showed significantly higher accumulation in AL at 26 DAA compared to 15 DAA, indicating that the TCA cycle is very active in AL during the late developmental stage to provide energy (Figure 3). Various amino acids accumulate during the grain filling (Zhang *et al*., 2021). In the present study, amino acid accumulation exhibited a particular pattern with significant changes between two developing time points (15 and 26 DAA) across the different cell types of the endosperm. For example, asparagine showed significant accumulation in SE and ETCs during 15 DAA and AL during 26 DAA. Asparagine serves for both the storage and transportation of nitrogen (Garg *et al*., 1984).

Similarly, at the proteome level, we identified higher regulation of nitrate metabolism in AL during 15 DAA. During grain filling, AL cells transport assimilates into endosperm (Zheng and Wang, 2014). The main function of ETCs is metabolite uptake, such as sugars and amino acids, for grain filling (Thompson *et al*., 2001). Hence, it can be concluded that N‐remobilization is highly active during 15 DAA compared to 26 DAA.

Interestingly, in 15 DAA, proline significantly accumulated in the inner endosperm compared with the outer endosperm (AL and SA; Figure 3). Proline is an osmotic agent and radical scavenger that protect cells from various abiotic stresses, which is important for actively dividing cells to maintain sustainable growth (Kavi Kishor and Sreenivasulu, 2014). Similarly, regulation of the protein pyrroline‐5‐carboxylate reductase (PyrCR), which plays an important role in proline synthesis, demonstrated higher regulation in all the endosperm cell types during 15 DAA compared to 26 DAA.

## Discussion

Wheat is the second most‐produced cereal globally, and it is an important source of food, feed and many industrial uses. Therefore, understanding the molecular mechanisms involved in grain development is important for breeding high‐quality wheat and achieving global food security. In our previous study, we analysed the dynamic changes in protein and metabolite levels in wheat grain at four sequential developmental stages, that is, 12, 15, 20, and 26 DAA, in four different components that is, seed coat, embryo, endosperm and cavity fluid (Zhang *et al*., 2021). Endosperm development is a highly complex process controlled by a grain‐filling mechanism. Eventually, endosperm composition defines the germination efficacy and nutritional quality of the seeds. In the present study, we investigated the different cell types (AL, SA, SE, ETCs) of the endosperm using the LMD approach to reveal the temporal and spatial distribution of proteins and metabolites at two important transition time points (15 and 26 DAA). So far, this is the first study where different cell types of developing wheat endosperm are studied using a multiomics approach.

Physiologically, the moisture content, fresh and dry weight of the wheat grain gradually increase from 12 DAA to 26 DAA (Zhang *et al*., 2021). After 10 DAA, grain completes the cellularisation and differentiation process and focuses on cell growth and elongation. Therefore, 15 and 26 DAA are considered the start and end of the fast accumulation stage during wheat grain development, in which cell content changes completely (Zhang *et al*., 2021). In the present study, the assimilates, such as soluble sugars, sucrose and starch content, demonstrated dynamic regulation during the grain‐filling process, indicating the essential role of sucrose metabolism and its association with the rate of starch accumulation (Figure 1e,f).

### Sugar loading in developing wheat endosperm

**Figure 4:**
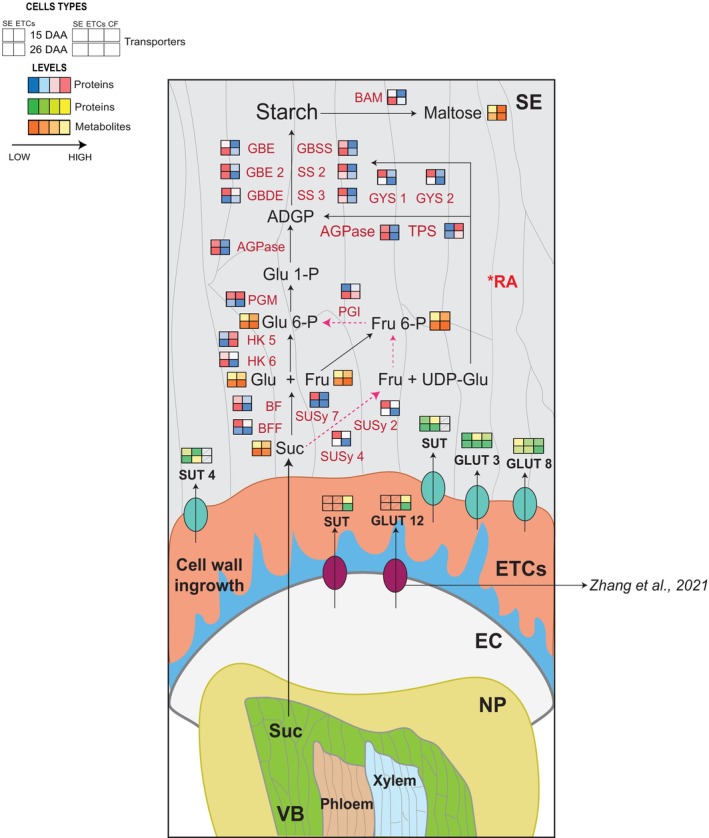
The conceptual model for the cellular route to sugar loading in wheat grain development. Metabolites and transporters are written in black letters. Proteins are written in red letters. Levels of transporters and enzymes were averaged over three biological replicates after normalization. Two‐row squares indicate 15 and 26 DAA. Proteins have two columns that indicate values in SE and ETCs, while transporters have three columns that indicate SE, ETCs and CF (CF: cavity fluid data obtained from Zhang *et al*., 2021). Values of protein levels from minimal to maximal are coloured from blue to red, and transporter levels are coloured from green to yellow. Metabolite levels from minimal to maximal are coloured from orange to yellow. AGPase, ADP‐glucose pyrophosphorylase; BAM, β‐amylase; BF, beta‐fructofuranosidase insoluble isoenzyme 2; BFF, 1 2‐beta‐fructan 1F‐fructosyltransferase; CF, cavity fluid; EC, endosperm cavity; ETCs, endosperm transfer cells; GBDE, glycogen debranching enzyme; GBE2, 1 4‐alpha‐glucan branching enzyme II; GBSS, granule‐bound starch synthase; GLUT, glucose transporter; HXK, hexokinase; NP, nucellar projection; PGI, phosphoglucoisomerase/glucose‐6‐phosphate isomerase; PGM, phosphoglucomutase; RA, redox activation; SE, starchy endosperm; SS, starch synthase; SUSy, sucrose synthase; SUT, sucrose transporter; TPS, trehalose‐phosphate synthase; VB, vascular bundles.

Starch biosynthesis in wheat grain is a complex process that includes the lysis of the endosperm cell wall, starch hydrolysis and transport of photoassimilates (sugars and amino acids). Sucrose is an important photoassimilate that originates from photosynthesis in source leaves. In this study, based on the proteomics and metabolomics analyses of the different cell types, we propose a model for the cellular route for effective sugar loading in developing wheat grain (15 and 26 DAA; Figure 4). Morphologically, sucrose transport in wheat is initiated via vascular bundles at the bottom of the crease. The crease vein is the site for phloem unloading, and sucrose is transported symplastically inwards and unloaded in the endosperm cavity (Thorne, 1985). Nucellar projection (NP) cells near to the endosperm develop cell wall ingrowth to facilitate sugar loading by increasing the cell surface (Figure 4; Wang *et al*., 1994; Zheng and Wang, 2011).

Further import of sucrose from the cavity fluid is facilitated with the help of two transporters (sucrose transporters (SUT) and glucose transporter (GLUT 12)) into the endosperm transfer cells (ETCs) during 15 DAA (Zhang *et al*., 2021). At the metabolome level, we also observed enhanced accumulation of sucrose in the inner endosperm (SE and ETCs) in 15 DAA compared with 26 DAA (Figures 3 and 4). From the ETCs, sucrose is loaded into the SE with the help of four SUT and GLUT transporters, and subsequently, sucrose is degraded to glucose and fructose by sucrose invertase (BF and BBF) as the first step for starch biosynthesis. Alternatively, sucrose is degraded to fructose and UDP‐glucose by sucrose synthase (SS). The accumulation pattern of glucose and fructose had a similar spatial distribution as sucrose in SE during the grain‐filling process (Figures 3 and 4). Rapid induction of the SUT and GLUT transporter correlates with increasing sucrose and SS activity during 15 DAA compared with 26 DAA, which occurs immediately before the onset of endosperm starch accumulation. It is also well correlated with the assimilation of sucrose during the early/mid‐grain filling stage (Figure 1c,d). The *TaSUT1* transporter was highly expressed during the mid‐grain filling stage of wheat and demonstrated a higher thousand‐grain weight and grain width and length with a 28% higher grain yield (Weichert *et al*., 2010). Hence, sucrose transport is extremely important for the developing grain in the early/mid‐stage. However, the regulation of the transporters varies between different cell types and the developing phase (15 and 26 DAA), which is essential for proper sucrose partitioning during the grain‐filling process (Figure 4).

### Starch metabolism in developing wheat endosperm

We identified all the key enzymes in the starch biosynthesis pathway, which include ADP‐glucose pyrophosphorylase (AGPases), two soluble starch synthases (SS), glycogen synthase (GYS) and granule–bound starch synthases (GBSSs). The activity of these enzymes strongly affects wheat grain weight (Figure 4). The regulation of all the key regulators increased during the early/mid‐grain filling stage (15 DAA) compared with the late grain filling stage (26 DAA). Two isoforms of SS and GYS demonstrated increased regulation during 15 DAA, which could contribute to the accumulation of amylose and amylopectin into starch granules during the early grain‐filling process (Figure 4). Among the identified starch biosynthesis proteins, five β‐amylase (BAM) isoforms were identified in all cell types (AL, SA, SE, ETCs) with increased regulation in SA and SE during 26 DAA compared with 15 DAA. β‐ amylase is a starch‐degrading enzyme that hydrolytically cleaves α‐1,4‐d‐glucosidic bonds to liberate β‐maltose from the non‐reducing ends of a variety of polyglucans that are synthesized during grain development. It is one of the major proteins in the starchy endosperm (SE). Similarly, at the metabolome level, we identified enhanced maltose accumulation in SE during 15 DAA compared with 26 DAA. Maltose is the major product of the degradation of linear glucans by the exoamylase β‐amylase (Schreier *et al*., 2021). Maltose accumulation can result in a high osmotic potential in early/mid‐developing grain (15 DAA; Figure 4), drawing in excess water that stretches the seed coat and pericarp. Loss of water during grain maturation leads to shrinkage when the grains mature, which can be related to the lower accumulation of maltose in the late grain filling stage (26 DAA; Schreier *et al*., 2021). Several studies have demonstrated that β‐amylase genes are transcribed and translated during barley grain development, with some enrichment in SA and AL (Betts *et al*., 2017; Radchuk *et al*., 2009). Most of the β‐amylase enzyme is in an inactive form before seed germination. Additionally, it has been reported that starch hydrolyzer enzymes (such as α‐amylase), except β‐amylase, are produced in AL tissue during embryo germination and then released to the SE (Hara‐Nishimura *et al*., 1986). During seed germination, a proteolytic mechanism activates the pre‐existing β‐amylase molecules in the SE (Beck and Ziegler, 1989; Shewry *et al*., 1988). Nadaud *et al*. (2015) suggested that the high abundance of β‐amylase in wheat AL may be related to the energy supply for the living AL cells. Furthermore, starch can also be temporally accumulated in AL cells, which can be degraded during the grain‐filling process and replaced by storage proteins and lipids. Therefore, the enhanced abundance of β‐amylase proteins in the AL is justifiable. All the proteins and metabolites discussed can be identified in Tables S10 and S11.

### Shifts in protein levels between aleurone (AL) and endosperm transfer cells (ETCs) (modified aleurone) during the grain‐filling process in wheat

After the photoassimilates are loaded into the developing endosperm from the endosperm cavity, AL and ETCs are two possible regulatory processes that regulate the spatial distribution of the starch and protein (Wang *et al*., 1995). Since ETCs are also called modified aleurone (Bechtel *et al*., 2009; Wang *et al*., 1995b), we explore the interaction of the AL and ETCs (Table 1). Two storage proteins (11S globulin seed storage protein and SSA1‐2S albumin seed storage family protein precursor) and protein translocase subunit SecA showed increased levels in AL during the grain‐filling process (change 1), indicating that AL plays an important role in protein storage. The proteins, which are highly abundant in ETCs during 15 and 26 DAA (change 2), were related to transporters (such as magnesium transporter MRS2‐1 and protein transport protein sec31) and enzymes (such as catalase, nitrate reductase and peroxidase 17; Table 1). This indicated that molecular transportation and biological processes were the key tasks of the ETCs in the grain‐filling process.

**Table 1:** Different protein distribution patterns in AL and ETCs

(a)	(b)						
	Protein Nr	Protein name	Change	15 AL	15 ETCs	26 AL	26 ETCs
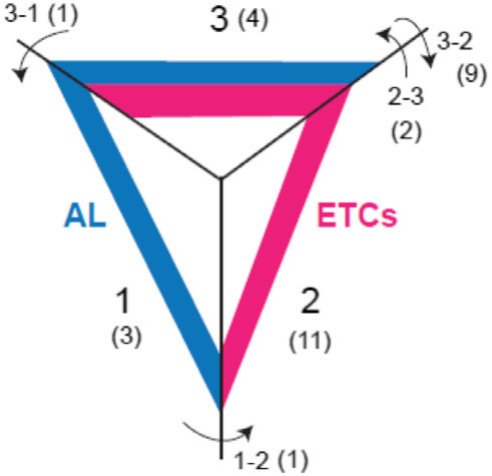	1	11S globulin seed storage protein (Fragment)	1	+	−	+	−
	2	Protein translocase subunit secA	1	+	−	+	−
	3	SSA1 ‐ 2S albumin seed storage family protein precursor, expressed	1	+	−	+	−
	4	Amino acid permease	2	−	+	−	+
	5	Catalase	2	−	+	−	+
	6	Disulfide isomerase‐like protein	2	−	+	−	+
	7	Glutamate synthase	2	−	+	−	+
	8	Glutathione‐regulated potassium‐efflux system protein	2	−	+	−	+
	9	Legumain/vacuolar processing enzyme	2	−	+	−	+
	10	Magnesium transporter MRS2‐1	2	−	+	−	+
	11	Mitochondrial 2‐oxoglutarate/malate carrier protein	2	−	+	−	+
	12	Nitrate reductase	2	−	+	−	+
	13	Peroxidase 17	2	−	+	−	+
	14	Protein transport protein sec31	2	−	+	−	+
	15	Alanine aminotransferase 2	3	+	+	+	+
	16	Disease resistance response	3	+	+	+	+
	17	Glutaredoxin C4	3	+	+	+	+
	18	Vicilin (Fragment)	3	+	+	+	+
	19	Vacuolar ATPase subunit H protein	1‐2	+	−	−	+
	20	PBSP domain protein	2‐3	−	+	+	+
	21	Transmembrane protein 205	2‐3	−	+	+	+
	22	Aminotransferase‐like protein	3‐1	+	+	+	−
	23	Glutathione synthetase	3‐2	+	+	−	+
	24	Mitochondrial import inner membrane translocase subunit tim50	3‐2	+	+	−	+
	25	MSP domain‐containing protein, putative, expressed	3‐2	+	+	−	+
	26	Oxysterol‐binding protein	3‐2	+	+	−	+
	27	Phosphatidylglycerol/phosphatidylinositol transfer protein	3‐2	+	+	−	+
	28	Potassium transporter	3‐2	+	+	−	+
	29	Transport protein SFT2	3‐2	+	+	−	+
	30	Vacuolar sorting‐associated protein	3‐2	+	+	−	+
	31	Vicilin‐like protein (Fragment)	3‐2	+	+	−	+

(a) The schematic figure describes the protein change. The blue band indicates AL; the pink band indicates ETCs. Change 1 means the protein abundant in AL, change 2 means the protein abundant in ETCs, and change 3 means the protein abundant in AL and ETCs. Arrows show the change during the grain filling, and the number beside the arrow indicates the change type (e.g. 1 changed to 2 during the grain filling marked as 1‐2). The numbers in parentheses indicate the number of proteins involved in different change types. (b) In the table, (+) indicates the high abundance, and (−) indicates the low or 0 levels of the protein.

Abbreviations: AL, aleurone; ETCs, endosperm transfer cells.

Magnesium (Mg^2+^) is a macro element required by all living cells. It serves as the central metal ion of the Chl molecule. It contributes to membrane stability, ion transport regulation, cation balance and the activation of many enzymes involved in various physiological processes (Shaul, 2002). As the mediator of the magnesium influx, magnesium transporter MRS2‐1 was identified in ETCs and kept a steady level during the grain filling, indicating that ETCs are the entry point for Mg^2+^, which is required during the grain‐filling process. Change 3 includes proteins with high abundance in AL and ETCs during the grain filling, including the alanine aminotransferase 2, disease resistance response, glutaredoxin C4 and vicilin (Table 1), which suggests that stress and redox may be the common functional connection between AL and ETCs.

Aminotransferase‐like protein was 3‐1, indicating that the active central amino acid metabolism changed from ETCs to AL during the grain filling. Only one or two proteins were 1‐2, 2‐3 and 3‐1, but 9 proteins were 3‐2, indicating that in 15 DAA, AL and ETCs were highly active, but ETCs were relatively more active in 26 DAA. The potassium transporter demonstrated the change 3‐2. Proteins involved in vesicle transport (MSP domain‐containing protein, putative, expressed and oxysterol‐binding protein) and vacuolar sorting (vacuolar sorting‐associated protein) were also changed (Table 1).

Seed‐type vacuolar processing enzymes are attributed to the maturation of storage proteins within protein storage vacuoles (Shimada *et al*., 2003; Wang *et al*., 2009; Zhang *et al*., 2021). Legumain/vacuolar processing enzyme was found in ETCs, indicating that ETCs are the pivotal endosperm cell type for protein processing (Table 1). The 11S globulin seed storage protein (fragment) showed an increased gradient from the ETCs to AL, which was strengthened during the grain‐filling process. Although the ETCs are the active site of protein processing, AL might show higher sink capacity (Table 1).

### Protein accumulation patterns during the developing wheat endosperm

#### Major changes of the aleurone (AL) during the endosperm development are related to carbohydrate metabolism, oxidative stress and signalling proteins

Through the process of cellularization, the interior cells become starchy endosperm. In contrast, the perimeter cells become aleurone, which reaches the maturity form at 14 DAA (Tosi *et al*., 2009) and remains quite active throughout the grain development process by building high metabolic activity. Here, we selected 15 DAA for our analysis to characterize this specific metabolic activity in the grain‐filling process. The protein and mineral accumulation in AL occurs between 11 and 27 DAA (Xiong *et al*., 2013; Moore *et al*., 2016).

Carbohydrate metabolism is fundamental to grain development; it is the main energy source. In the present study, five proteins were down‐regulated in AL out of six in 15 and 26 DAA involved in the glycolysis pathway. Most proteins associated with the C3 cycle/gluconeogenesis also showed down‐regulation in the AL in both 15 and 26 DAA, while proteins related to glucose metabolism demonstrated the opposite trend (Table S9). During AL development, the up‐regulation of glucose‐6‐phosphate isomerase and two isoforms of hexokinase 6 suggest their important roles in achieving desiccation tolerance by accumulating sugars with osmo‐protective activity (Witzel *et al*., 2010; Figure S2). They are essential enzymes in glucose metabolism (Dupont, 2008). Interestingly, similar regulation of carbohydrate metabolism was observed in the early stages of wheat grain development (Nadaud *et al*., 2015). The AL is the peripheral grain tissue rich in metabolites such as micronutrients, vitamins, antioxidants and essential amino acids (Nadaud *et al*., 2015). Hence, it further confirms the hypothesis that AL is upstream of the metabolic processes and assimilates the transport pathway in the endosperm.

Oxidative stress proteins (OS) have an important role during the grain‐filling process; they it facilitate the production of amino acids and control ROS activity, which enables the accumulation and maturation of globulins within the AL. Oxidative stress (OS) and defence proteins protect AL accumulation during grain development (Nadaud *et al*., 2015). These results are consistent with our observation, where we observed increased oxidative stress proteins (such as glutathione transferase, aquaporin, elongation factor beta‐1) in the AL during the grain filling stages (15 and 26 DAA; Table S9). Similarly, the regulation of signalling proteins was also significant only in the AL (Table S9). 14‐3‐3 proteins play a regulatory role in various cellular physiological processes, such as cell signal transduction, cell cycle regulation and nitrogen and carbon assimilation (Ferl, 1996; Fulgosi *et al*., 2002). Nadaud *et al*. (2015) observed that four 14‐3‐3 proteins were equally abundant throughout AL development, as signalling and signal transduction are maximum during the early stages and decrease during the mid‐stage of grain development. A decrease in 14‐3‐3 is inversely related to starch synthesis and accumulation (Zhang *et al*., 2014). However, in the present study, no significant change in the regulation of 14‐3‐3 protein was observed in SA and SE (Figure S2).

#### Stress/defence proteins are activated in the starchy endosperm (SE) during the endosperm development

Stress/defence proteins play a critical role in protection during the storage accumulation phase. Nadaud *et al*. (2015) reported that among the three main stages of grain development, the mid‐stage is characterized by oxidative stress and defence proteins (65%), which can be linked with water loss in peripheral layers during grain development. Interestingly, serpin (serine protease inhibitor) proteins were identified among stress/defence proteins with significantly high abundance in all the cell types during 26 DAA compared with 15 DAA (Table S9). Serpin proteins are highly expressed during seed maturation and protect cells from oxidative stress during development (Roberts and Hejgaard, 2008). Additionally, it was found that wheat serpins may play a protective role for prolamins (Ostergaard *et al*., 2000), which are available around the amyloplasts. They probably protect starch in SE tissue during the endosperm development (Figure S2). These proteins also protect the cellular contents of dehydrated grain (Tasleem‐Tahir *et al*., 2012). Previously, we have reported that regulation of serpin proteins had increased in all tissues of wheat grain (i.e. seed coat, cavity fluid and endosperm) except for embryo during 12–26 DAA (Zhang *et al*., 2021). Additionally, several other proteins were identified with significantly different accumulation patterns in the different cell types during 15 and 26 DAA (Figure S2, Table S9).

#### Programmed cell death (PCD) in developing wheat endosperm

**Figure 5:**
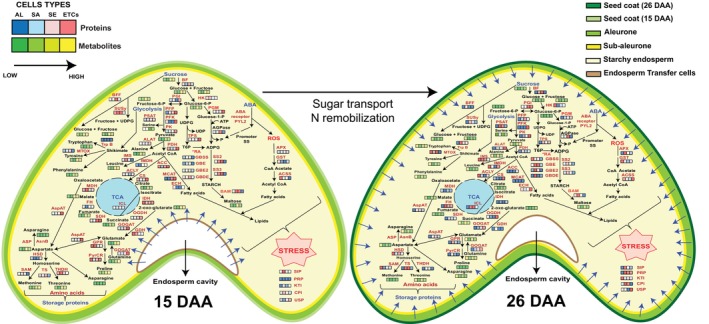
Biochemical pathway map for proteins and metabolites dynamics in different endosperm cell layers during 15 and 26 DAA. Metabolites are written in black letters; proteins are written in red letters. Levels of proteins and metabolites were averaged over three replicates after normalization. Four consecutive squares from left to right indicate four cell types of the endosperm (AL, aleurone; ETCs, transfer cells; SA, sub‐aleurone; SE, starchy endosperm) in 15 and 26 DAA. Values of metabolite levels from minimal to maximal are coloured from green to yellow, and values of protein levels from minimal to maximal are coloured from blue to red. ACC, acetyl‐CoA carboxylase; ACLY, ATP citrate lyase; ACSS, acyl‐CoA synthetase; AGPase, glucose‐1‐phosphate adenylyltransferase; ALAT, alanine aminotransferase; APX, ascorbate peroxidase; AspAT, aspartate aminotransferase; BAM, beta‐amylase; BF, beta‐fructofuranosidase insoluble isoenzyme 2 (Cell wall invertase); BFF, 1 2‐beta‐fructan 1F‐fructosyltransferase (Vacuolar invertase, VINV); CPI, cysteine proteinase inhibitor; CS, citrate synthase; ECH, enoyl‐CoA hydratase; FH, fumarate hydratase class II; GBDE, glycogen debranching enzyme; GBE, 1 4‐alpha‐glucan branching enzyme; GBE2, 1 4‐alpha‐glucan branching enzyme II; GBSS, granule‐bound starch synthase; GDH, glutamate dehydrogenase; GOGAT, glutamate synthase; GPR, gamma‐glutamyl phosphate reductase; GST, glutathione S‐transferase; HK, hexokinase; HSD, aspartokinase/homoserine dehydrogenase; ICL, isocitrate lyase; IDH, isocitrate dehydrogenase; IMDH, 3‐isopropylmalate dehydrogenase; IMS, 2‐isopropylmalate synthase 1; KTI, Kunitz trypsin inhibitor; MCAT, malonyl CoA‐acyl carrier protein transacylase; MDH, Malate dehydrogenase; MTOX, N‐methyl‐L‐tryptophan oxidase; OGDH, 2‐oxoglutarate dehydrogenase; PDH, pyruvate dehydrogenase E1; PFK, phosphofructokinase family protein; PFP, diphosphate‐fructose‐6‐phosphate 1‐phosphotransferase; PGI, phosphoglucoisomerase/glucose‐6‐phosphate isomerase; PK, pyruvate kinase; PRP, pathogenesis‐related protein 4B (Fragment); PSAT, phosphoserine aminotransferase; PyrCR, pyrroline‐5‐carboxylate reductase; SAM, S‐adenosylmethionine synthase; SDH, succinate dehydrogenase; SIP, stress‐inducible protein; SS2, Starch synthase 2; SS3, soluble starch synthase 3; SUSy, sucrose synthase; THDH, threonine dehydratase biosynthetic; TPS, trehalose‐phosphate synthase; TrpB, tryptophan synthase beta chain 1; TS, threonine synthase; USP, universal stress protein.

Given that active gene expression and functional organelles are necessary for both starch biosynthesis and storage protein accumulation, Liu *et al*. (2022) proposed a highly specialized form of PCD in starchy endosperm cells, wherein only the cytoplasmic membrane loses integrity, while most intracellular organelles, such as nuclei, mitochondria, plastids, endoplasmic reticulum (ER) and Golgi apparatus, remain functional and intact. This forms a large compartment within the shared cytoplasm, allowing sugars and amino acids to circulate freely and enabling effective starch biosynthesis and storage protein accumulation (Liu *et al*., 2022; Xu *et al*., 2016). Gradients of cell death can be observed during the development of endosperm, which exhibit species‐specific patterns. In maize endosperm, cell death moves from the crown towards the base. In barley seeds, PCD occurs in a gradient manner from central to peripheral regions (Sreenivasulu *et al*., 2006; Young and Gallie, 2000). Conversely, in wheat endosperm, PCD in starchy endosperm also starts at 16 DAA and lasts to 30 DAA; the occurrence of cell death is random, lacking a clear‐cut wave‐like pattern (Radchuk *et al*., 2011; Van Hautegem *et al*., 2015). In the present study, PCD, which involves glycolate oxidase, was identified in all endosperm cell types. The abundance remained generally stable among the endosperm cell types and different grain‐filling stages, with relatively higher levels in SE and ETCs at 26 DAA (Table S7). Moreover, there was no pattern to the abundance of abscisic acid receptor PYL2 in different endosperm cell types during the grain filling (Table S7). Reactive oxygen species (ROS), including hydrogen peroxide (H_2_O_2_), superoxide anions ($O_{2}^{-}$), hydroxyl radical (·OH) and singlet oxygen (O_2_), are metabolic by‐products which are associated with the initiation, progression and regulatory orchestration of PCD in plants (Cheng *et al*., 2016). Plants have developed a robust defence against the harmful impact of reactive oxygen species (ROS) through the deployment of an antioxidant enzyme system (including superoxide dismutase, catalase and peroxidase) as well as antioxidant compounds (like glutathione). These components collectively neutralize and effectively eliminate ROS‐induced cellular injury (Cheng *et al*., 2016; Li *et al*., 2018). The superoxide dismutase was identified in SA at 26 DAA (Table S7), while catalase was found in all endosperm cell types during the grain‐filling process, with significantly high levels in ETCs (Table 1 and Table S7). Peroxidase was abundant in SA and SE at 26 DAA, while peroxidase 17 showed a high level in ETCs and peroxidase 65 in SE at 26 DAA (Table 1; Table S7). Other enzymes involved in ROS and stress systems are shown in Figure 5.

#### Proteome and metabolome landscape in different cell types of developing endosperm

To better understand the biochemical mechanisms, as well as related metabolome and proteome changes in different cell types of wheat endosperm (AL, SA, SE and ETCs), putative metabolic pathways are proposed (Figure 5). The main pathways involved in different cell types with different regulations are carbohydrate metabolism (TCA cycle, glycolysis, starch synthesis), storage protein, amino acid metabolism, lipid metabolism, stress/defence protein and oxidative stress/redox. Overall, TCA/Gluconeogenesis and amino acid metabolism demonstrated a gradual decrease from 15 DAA to 26 DAA, indicating that primary metabolism in different cell types of wheat endosperm is reduced or changed to metabolic pathways with less ATP consumption during the late stage of grain development. The molecules, such as sucrose and amino acids, are first synthesized during early/mid‐stage and are used for starch and storage protein synthesis during endosperm development (Figure 5). Other important metabolic processes during endosperm development involve stress defence and oxidative stress proteins/redox to guarantee normal endosperm development during the late stage of development.

The energy demands of germinating seeds seem to be met mainly by glycolysis (Yang *et al*., 2007). In the present study, essential proteins involved in glycolysis, including diphosphate‐fructose‐6‐phosphate 1‐phosphotransferase (PFP), phosphofructokinase family protein (PFK) and pyruvate kinase (PK), showed increased levels in ETCs during 15 and 26 DAA. At the metabolome level, glycolysis‐related metabolites (glucose‐6‐phosphate, fructose‐6‐phosphate and glycerol‐3‐phosphate) showed less regulation in ETCs than SE. In contrast, the pyruvate levels (the glycolysis product) demonstrated increased levels in ETCs (Figure 5). These findings indicate that ETCs are important in supplying energy (ATP) to the developing endosperm.

Aspartate aminotransferase (AspAT) and alanine aminotransferase (ALAT) are essential enzymes for metabolic intermediates. They are important substrates for protein synthesis, which is directly linked to the TCA and amino acid metabolism. Aspartate and alanine may also supplement the glutamate pool as the role of amino group donors (Gajewska and Sklodowska, 2009). Alanine aminotransferases (ALAT) were up‐regulated in the ETCs during 15 and 26 DAA. Phosphoserine aminotransferase (PSAT) is another important enzyme in TCA and amino acid metabolism. PSAT showed increased regulation in SA and SE during 26 DAA compared with 15 DAA, which guaranteed the supply of glutamate during the grain‐filling process.

Proteins related to stress metabolism significantly increased during 26 DAA, especially universal stress protein (USP) in AL and ETCs. USP proteins of plants are involved in responses to various abiotic stressors and biopathogens. However, in seed development, they might regulate other physiological processes, such as hormone regulations. In this study, we also identified several heat shock proteins (HSPs), including small heat shock proteins (sHSPs), which might be involved in proper protein folding and act as molecular chaperones under stress conditions to protect the developmental process.

The spatial distribution of protein content and quality in the mature wheat grain has been determined. As discussed, two pivotal enzymes involved in glucose metabolism (glucose‐6‐phosphate isomerase and hexokinase 6) exhibited up‐regulation in AL (Figure S2) and play an important role in accumulating sugars with osmo‐protective activity against desiccation tolerance. The metabolism of carbohydrates and storage proteins in different endosperm cell types intricately interact and regulate each other, ultimately enabling each endosperm cell type to exhibit distinct functions and regulations. The forming process of this spatial distribution in mature wheat grains has been speculated (Xurun *et al*., 2015), and it is based on the two transport pathways during the grain‐filling process. One is transported to the inner endosperm from the AL; the other is through the ETCs around the endosperm cavity radially to the endosperm (Figure 5). It is unclear which pathway plays a major role in different grain‐filling stages. It is postulated that these two pathways change according to the different grain‐filling stages (Moore *et al*., 2016; Xiong, 2013). However, in the present study, we suggest that the two pathways are switched due to the accumulation of different assimilates and regulation of proteins and metabolites (see ‘Sugar loading and starch biosynthesis in developing wheat endosperm’ and Figures 4 and 5). The cooperative regulation of different cell types in the endosperm provides new insights into the dynamic interplay of the proteome and metabolome during wheat endosperm development. All the proteins and metabolites discussed can be identified in Tables S10 and S11.

## Conclusion

Aleurone, sub‐aleurone, starchy endosperm and endosperm transfer cells comprise the wheat endosperm, each with its own spatial–temporal physiological and molecular mechanism. All these distinct cell types develop at different rates. In this study, 1803 proteins and 41 metabolites were identified, and the results showed that the accumulation patterns of these proteins and metabolites vary in different endosperm cell types during development. For example, AL demonstrated a highly differentially expressed proteome and metabolome, which is distributed between carbohydrate biogenesis, oxidative stress and signalling. Although previous studies have identified some individual genes encoding sugar transporters and enzymes involved in sugar unloading and partitioning in wheat grains (Aoki *et al*., 2004, 2006; Liu *et al*., 2021), there is a lack of a holistic understanding of the molecular landscape governing sucrose transport and utilization in developing endosperm, which includes the interplay of each cell type. For example, little is known about the role of SUT and GLUT transporters during the early/mid stages of grain filling, which initiates the starch biosynthesis in the grain‐filling process. Similarly, in the present study, an interesting cell type‐specific interplay of GOGAT, GDH and glutamic acid was determined over the course of development. GDH incorporates ${\mathrm{NH}}_{4}^{+}$ into 2‐oxoglutarate to form glutamate or oxidize glutamate. Although the 15 N‐ or 13C‐labeling experiments have demonstrated that the deamination reaction occurs in the cell, it has been argued that under certain physiological conditions, when the ${\mathrm{NH}}_{4}^{+}$ concentration reaches a certain threshold, the GDH is involved in ${\mathrm{NH}}_{4}^{+}$ assimilation (Dubois *et al*., 2003). So, it will be interesting to study the role of GS, GOGAT and GDH in grains with a high concentration of ${\mathrm{NH}}_{4}^{+}$ in different cell types of the developing endosperm.

Similarly, nitrate metabolism, nitrate transport, ABC transporters and multidrug resistance systems showed increased regulation in the outer endosperm (AL and SA) during the early/mid grain filling stage (15 DAA) and then in the inner endosperm (ETCs) during late developmental stage (26 DAA). Additionally, our metabolome approach identified dynamic changes in metabolite levels and correlations among different cell types in developing endosperm. This study provides the framework for future analysis to understand the spatial distribution of proteins and metabolites during grain development, which can be improved according to the breeding goals.

## Conflict of interest

The authors declare no competing financial interest.

## Funding

S.Z. was supported by the China Scholarship Council (CSC) (Grant number: 201706850037) and completion grant of the Vienna Doctoral School Ecology and Evolution of the Faculty of Life Sciences, University of Vienna, Austria. H.K. is supported by the doctoral programme MENTOR (Molecular Mechanisms to Improve Plant Resilience), funded by the Austrian Science Fund (FWF) under project number DOC 111. A.G. is thankful to the European Union Horizon 2020 research and innovation programme under grant agreement number GA 2020 862–858 (ADAPT). P.C. is thankful to the Austrian Science Fund (FWF, DerWissenschaftsfonds), grant agreement number I 5234, for their support.

## Author contributions

Conceived and designed the experiments: SZ, PC and WW. Performed the experiments: SZ, AG and PC. Performed LC–MS analysis: PC and SZ. Performed GC–MS analysis: PC, SZ and AG. Analysed the data: SZ, PC, AG, MMB, HK, FZ, SG, ZR, KG, DJ, RKV, WW. Drafted the manuscript: PC, AG, SZ and WW. Designed the figures: PC. Edited the manuscript: PC and WW. All the authors read and agreed on the final version of the manuscript.

## Supporting information


**Figure S1** Functional distribution of the proteome identified in different cell types of the developing wheat endosperm (15 and 26 DAA).
**Figure S2** Bar graph represents the accumulation pattern of the selected proteins.

**Table S1** Details of the morphological parameters (fresh weight, length and width of the grain), sucrose, soluble sugar and starch content measured during the wheat grain development.

**Table S2** Information of the identified protein candidates from different developing stages (15 and 26 DAA) in the endosperm cell types using Uniprot and IWGSC databases.

**Table S3** List of selected protein candidates: Proteins that are presented in all three biological replicates at least in one of the cell types in different developing stages (15 and 26 DAA) (max count = 3).

**Table S4** PCA loadings of identified protein candidates from different cell types during developing stages (15 and 26 DAA).

**Table S5** Venn analyses the identified protein candidates from different developing stages (15 and 26 DAA) in aleurone (AL), sub‐aleurone (SA), starchy endosperm (SE) and endosperm transfer cells (ETCs).

**Table S6** Differentially expressed proteins (DEPs): Proteins showing 1.5‐fold expression level and *P*‐value ≤ 0.05 between AL 15 vs AL 26 DAA, SA 15 vs SA 26 DAA, SE 15 vs SE 26 DAA and ETCs 15 vs ETCs 26 DAA in the different cell types during developing stages (15 and 26 DAA).

**Table S7** Functional categories of protein candidates were identified in the different cell types during the developing stages (15 and 26 DAA).

**Table S8** K – means cluster analysis of the identified protein candidates from different cell types during 15 and 26 DAA.

**Table S9** Protein accumulation pattern of the selected functional categories in aleurone (AL), sub‐aleurone (SA), starchy endosperm (SE) and endosperm transfer cells (ETCs) during developing stages (15 and 26 DAA).

**Table S10** GC‐TOF‐MS data from different cell types (AL, SA, SE, ETCs) of developing wheat endosperm (15 and 26 DAA).

**Table S11** List of proteins discussed in figures 4 and 5.

## Data Availability

The proteome data and raw files were deposited in the PRIDE repository with accession no. PXD035848.
