# Supplementary material for: Cell‐type proteomic and metabolomic resolution of early and late grain filling stages of wheat endosperm
Source: Plant Biotechnol J. 2023 Dec 4;22(3):555–71. doi: 10.1111/pbi.14203 (PMC12047074; doi:10.1111/pbi.14203)
Supplement: Supplementary file 1 — Figure S1 Functional distribution of the proteome identified in different cell types of the developing wheat endosperm (15 and 26 DAA). Figure S2 Bar graph represents the accumulation pattern of the selected proteins. [file PBI-22-555-s007.pptx]

## Slide 1
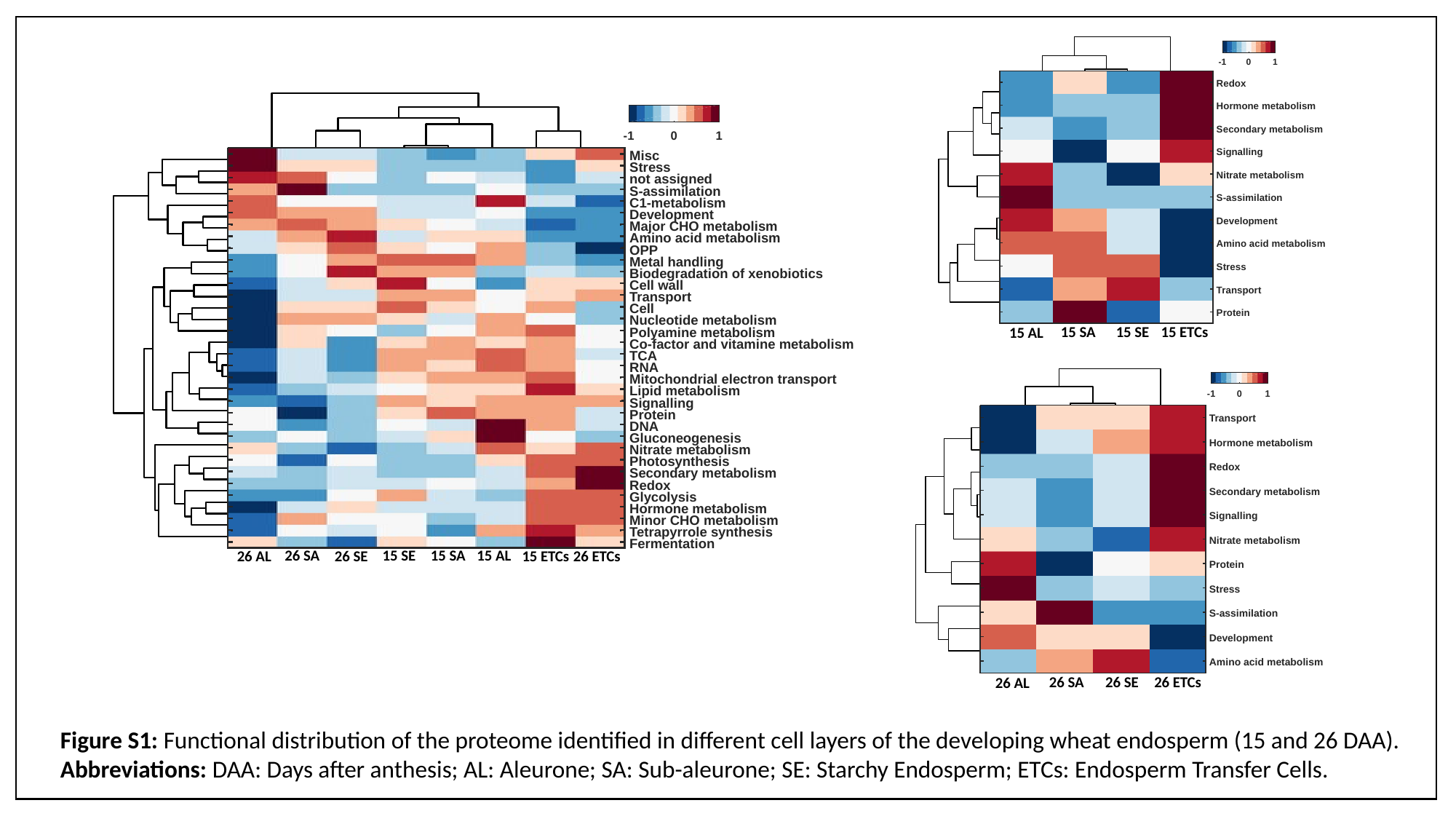

15 SA
15 SE
15 ETCs
15 AL
15 SE
15 AL
15 SA
26 SA
26 AL
26 SE
15 ETCs
26 ETCs
26 SA
26 SE
26 ETCs
26 AL
Figure S1: Functional distribution of the proteome identified in different cell layers of the developing wheat endosperm (15 and 26 DAA). Abbreviations: DAA: Days after anthesis; AL: Aleurone; SA: Sub-aleurone; SE: Starchy Endosperm; ETCs: Endosperm Transfer Cells.

## Slide 2
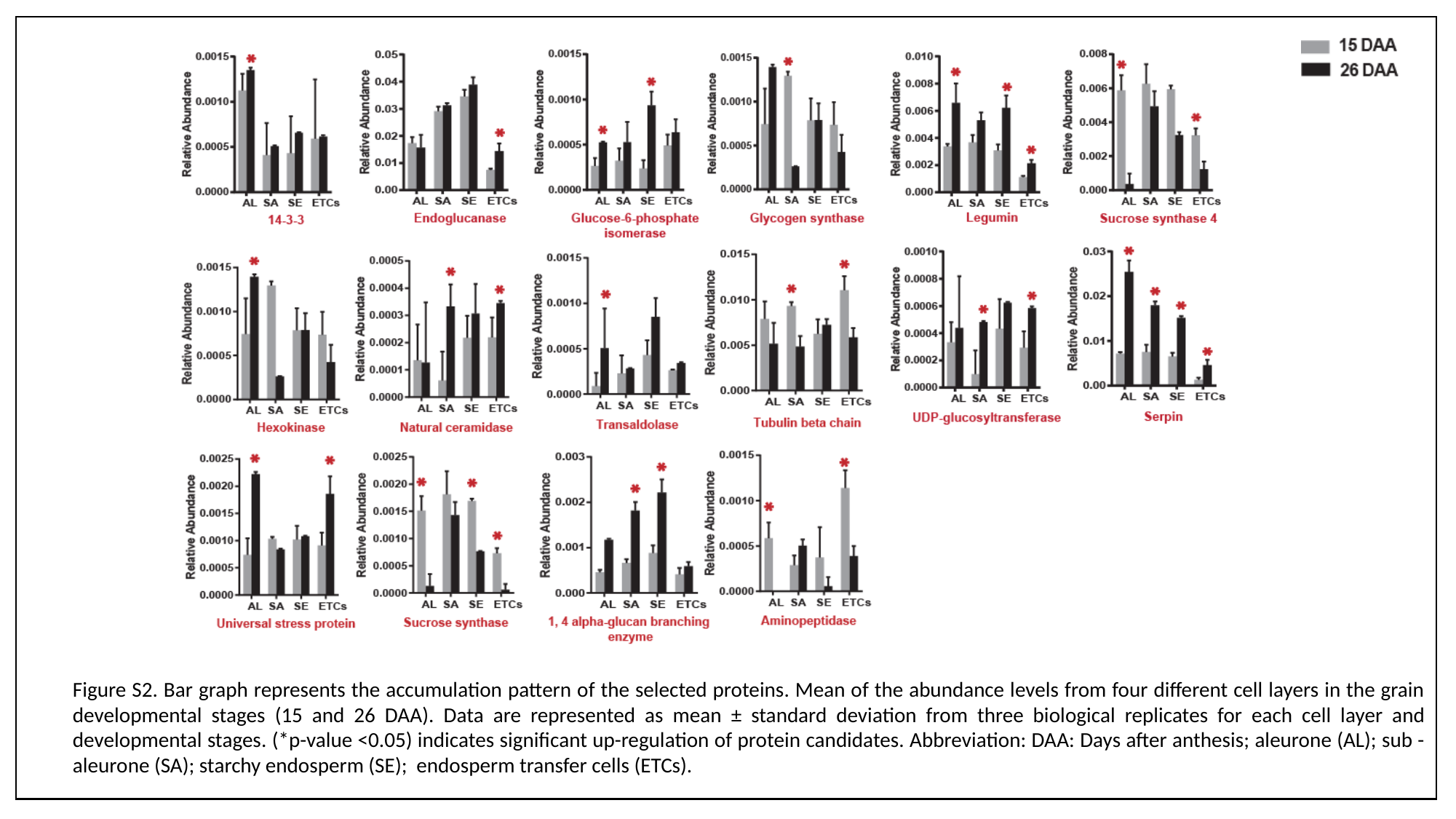

Figure S2. Bar graph represents the accumulation pattern of the selected proteins. Mean of the abundance levels from four different cell layers in the grain developmental stages (15 and 26 DAA). Data are represented as mean ± standard deviation from three biological replicates for each cell layer and developmental stages. (*p-value <0.05) indicates significant up-regulation of protein candidates. Abbreviation: DAA: Days after anthesis; aleurone (AL); sub -aleurone (SA); starchy endosperm (SE); endosperm transfer cells (ETCs).
